# Assessing and managing the suicidal patient: forget the Reverend Bayes and try game theory

**DOI:** 10.1192/bjb.2024.76

**Published:** 2025-06

**Authors:** Olav Nielssen

**Affiliations:** Macquarie University, Sydney, New South Wales, Australia

**Keywords:** Suicide, risk assessment, probability, game theory

## Abstract

Probability-based estimates of the future suicide of psychiatric patients are of little assistance in clinical practice. This article proposes strategic management of the interaction between the clinician and the patient in the assessment of potentially suicidal patients, using principles derived from game theory, to achieve a therapeutic outcome that minimises the likelihood of suicide. Further developments in the applications of large language models could allow us to quantify the basis for clinical decisions in individual patients. Documenting the basis of those decisions would help to demonstrate an adequate standard of care in every interaction.

The Reverend Thomas Bayes (1701–1761) is credited with devising a formula for calculating the probability of an event based on prior knowledge of conditions that might be related to the event. When applied to suicide risk assessment, Bayes theorem allows estimation of the sensitivity, specificity and positive predictive values of risk assessment models derived from studies of combinations of known risk factors, which in the case of suicide might include previous suicide attempts, psychiatric hospital admissions, disclosed suicidal thoughts and plans, and literally hundreds of other potential risk factors. Statistical analyses based on machine learning, in which the weights given to additional risk factors are constantly recalculated according to known associations with suicide in studied populations, have improved probability estimates for suicide, and known risk factors have been incorporated into suicide risk assessment procedures that are widely accepted by clinicians, taught to medical students, form part of the procedures of health services, are official government policy and are central to mental health law.

Despite access to very large and detailed data-sets that can be used to refine suicide risk models,^1,2^ the utility of applying estimates to an individual patient is quite limited because of the overwhelming numbers of false positives generated by even the latest statistical techniques, as well as the inevitability of suicides among patients classified as lower risk.^3^ Even the notion of a low-risk patient is of limited utility, as the probability of suicide in the next year of any patient discharged from a psychiatric ward,^4^ or from an emergency department after a suicide attempt,^5^ is as much as a hundred times greater than the rate of suicide in the wider community, whereas the differences in the odds ratios derived from probability are at their very best about tenfold.^6^ In other words, there is no such thing as a low-risk patient.^7^

We assume that the risk of suicide carries a fixed conditional probability, although that probability is updated after the suicide of one or more patients with the same risk factors. We also know there are limits to probability theory in complex systems, in which unpredicted events intervene and sometimes have a magnifying effect, as described by chaos theory.

A large part of the workload of mental health services is the assessment of and care for patients who disclose thoughts of suicide. Services also face lawsuits for failing to anticipate and prevent the suicide of patients discharged to community care. To improve the performance of services, this paper suggests applying principles of game theory, which models strategic interactions in which the other person's responses affect the outcome. The ‘game’ is the interaction between two interdependent participants, in this case the clinician and the patient, whose decisions are guided by what they understand to be their own best interests. Game theory began as a mathematical discipline, originally applied to the field of economics and later adapted to military strategy, but it is just as relevant to social sciences. The use of principles of game theory has been proposed in medical education,^8^ surgical units^9^ and transcultural evaluations^10^ and to enhance service cooperation.^11^ However, decisional science has not yet been formally applied to evaluating and managing potentially suicidal patients. Given our inability to usefully predict suicide using probability estimates,^12^ an alternative approach is to employ the principles of game theory to better understand the motivations of and decisions made by patients, in order to provide an adequate standard of care to each individual patient and to reach satisfactory outcomes for both patients and the mental health services charged with caring for them.

## Suicide risk assessment scenarios

Consider this situation. You are a trainee psychiatrist on call at a hospital the day after New Year. After a quick tour of your ward to help decide which patients will remain on close observation and which patients will be granted day leave, you head to the emergency department to interview the patients recovering from various forms of self-harm inflicted the previous night. There you find four patients have self-harmed, against the background of a range of intractable social problems, recurrent mental health crises, dissatisfaction with health services and failure of support networks. You know that you can find at best two beds for overnight observation, if a partly recovered patient can somehow be discharged. You also know that by their very presence in the emergency department, the probability that any one of these patients will die by suicide in the next year is many times greater than it was the day before. How can game theory assist in this situation?

The first premise of game theory is that the interaction between the players, in this case the clinician and the patient being assessed, is between two rational decision makers, which of course cannot be assumed in people with mental disorder. Nevertheless, a good starting point is Nielssen's first law of forensic psychiatry, that psychopaths always act in their own perceived best interest, facetiously proposed to remind trainees that disturbed conduct in prison that cannot be understood from the prisoner's perspective is likely to be due to some kind of mental illness. In other words, consider the perspective of the patient – what do they want from the interaction, and how might their needs be met? In pathologically irrational patients, such as those affected by evolving delusional beliefs, or those who have a gross disturbance of their capacity for logical thinking because of confusion, the solution is fairly simple. Those two beds will be needed. For the others, game theory can be applied to evaluate their needs and reach short-term and longer-term solutions, ideally to the satisfaction of the patient and their support network.

## Strategies and types of games relevant to clinical assessment

Strategies for the clinician are the range of approaches to intervention and communication, whereas strategies for the patient are the disclosure or non-disclosure of information, and cooperation with assessment and any proposed interventions. The payoff for the clinician is relatively straightforward: the success or failure of the intervention or outcomes more generally. The objective of the game is to maximise the outcome for the patient and hence the service. The payoff for the patient might be less clear, as a patient with a mental illness or in acute distress cannot be assumed to be acting rationally and may also be ambivalent in their wishes and intent. However, their decision-making can be modelled from attempts to understand their perception of the situation, and the optimal outcome for the suicidal patient is often to receive effective treatment that improves decision-making and resolves their real and perceived difficulties.

### Cooperative and non-cooperative games

An example of a cooperative game is a care-seeking patient and a clinician in a position to arrange suitable care. In those situations, being admitted to hospital or receiving some other assistance after suicidal behaviour or disclosing suicidal thoughts can provide a solution to the patient's problem. The patient discloses a plan to commit suicide, and the clinician, with the support of other services, does their best to convince the patient the plan is irrational and to reach an agreement with the patient to abandon threats of suicide and participate in an ongoing treatment plan drawn up together. An example of a non-cooperative game might be a patient who conceals information or who threatens to commit suicide unless the clinician is able to bring about an outcome that might be beyond the clinician's control, for example, the prescription of potent opioids, or the custody of a child. However, the knowledge that the patient would not commit suicide if certain conditions were met presents a starting point for sympathetic negotiation. The original description by Von Neumman and Morgenstern is of a cooperative game in which the players’ decisions minimise their own losses, and they make binding agreements to maximise their gain, although Von Neumann noted the effect of human agency on the desired outcome and on players’ decisions. John Nash, himself no stranger to mental health services, noted that lack of cooperation often results in failure to achieve the best outcome and observed that if the best outcome is not achieved, it is because at least one player needs to be educated on how to more effectively pursue their own interests. The Nash equilibrium is a solution to a non-cooperative game in which there is no incentive for a player to deviate, because the other player does not make any concessions.

### Symmetric and asymmetric games

The interaction between a clinician and a suicidal patient is usually an example of an asymmetric game, in which the patient is at a low point in their life and cannot see the alternatives open to them, whereas the clinician is seen as a person with greater perspective and experience of observing other people in similar situations, as well as having the legal authority to enforce protective care. The information available is also usually asymmetric, as the patient knows their full history and circumstances, whereas the assessing clinician will invariably have less information. Although the desired outcome for both players is the survival of the suicidal patient, the desired pathway to that outcome, and hence the resources required to reach it, can be quite different.

### Zero sum games

In zero sum games, a gain by one player – either admission or discharge from hospital, as the case may be for the patient – may be a loss for the other – for the clinician, in the form of allocation of a precious observation bed or the catastrophe of the suicide of a potentially treatable patient. In this scenario, the task of the clinician is to attempt to discover the patient's true intention, in the same way a poker player might guess at the commitment to a hidden card.

### Dynamic games

The assessment of suicidal patients can involve changes in circumstances, for example, returning to sobriety or communication from a rejecting partner, and these are also part of the dynamic reassessment with every new crisis in the lives of well-known patients who are known to carry a chronic risk of completed suicide. The objective of the game for the clinician might be to engage the patient in longer-term care that addresses fluctuations in mood state without hospital admission, which can in itself be harmful for highly changeable patients who also carry a chronic risk of suicide.

### Players’ decisions based on imperfect information

This scenario is the most common situation in the cross-sectional assessment of a suicidal patient, who might have a complex history that a busy clinician may not have time to review. There is likely to be information about the circumstances of self-harm or the patient's intention that cannot be known to the clinician but might appear in the coroner's brief, such as the nature of the patient's internet searches. The lesson in this situation is for the clinician to efficiently review the history and obtain all possible corroborative information in order to level the playing field, so to speak, and to be able to document the basis for decisions by documenting awareness of history and also gaps in the available information.

### Cooperative versus dominant strategy

Most clinical interactions employ a cooperative strategy, especially when patients are deemed to be capable of making informed decisions about their healthcare based on the information provided to them and selecting from the options offered to them by the clinician. Dominant strategies refer to a strategy that is considered optimal regardless of the strategies chosen by the other player and includes following set protocols that do not require cooperation, for example, involuntary admission to hospital.

## To be or not to be – that is often the question

An assumption in the application of game theory is that the decisions made by the other person are rational. Hence, we assume that at some level at least, the patient does not want to die, as human instinct is to survive despite the odds. However, a distressing reality is that among our many care-seeking patients are a number who view suicide to be a rational solution to intolerable or insoluble problems, either because these problems are real, such as bankruptcy, humiliation or rejection, or because of the morbid view of the world and oneself that accompanies severe depression. We assume that suicidal behaviour is a better predictor of an intention to commit suicide than disclosed intention. The failure of disclosed suicidal thoughts to reliably identify people who go on to commit suicide^13^ must be partly due to a small number who conceal those thoughts because of the implications of doing so during a clinical assessment in which the clinician has the power to invoke mental health law or because they simply have no confidence in the clinician to change the situation. Forecasts based on the assumption that the other person will behave rationally may not apply to patients who are already determined to commit suicide, and the probability of suicide in those patients, especially in the longer term, may not be modifiable.^2^

## Counter countertransference

One aspect of patient assessment that has been overlooked during the reductionist and biological era of psychiatry has been the importance of countertransference in clinical interactions, as a tool to both recognise the nature of the other person's problems and also minimise the harmful effects of any reactions by staff and the service. The core pathology of many self harming patients, who may have ambivalent intent and yet carry a much greater likelihood of eventual suicide, is fear of rejection. Despite this, their fear is often fulfilled through the antipathy created by their self-harming and care-seeking behaviour. Interactions with the staff of health services can determine whether the patient will respond to an appeal to reason and cooperate with a treatment plan; hence, the experience of clinical care may affect decisions made after discharge. The successful application of game theory to these patients requires maintaining professional objectivity and adopting a shared strategy to win over unpopular and manifestly self-defeating patients.

## Applying principles of game theory to potentially suicidal patients

**Fig. 1:**
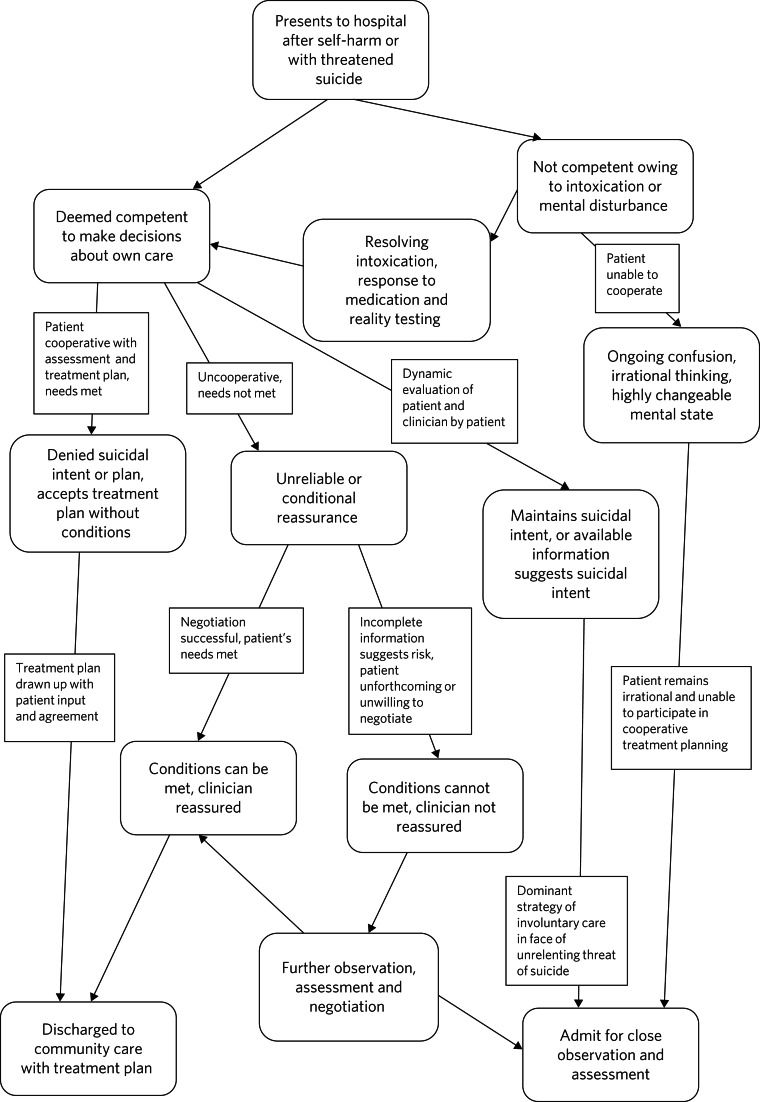
Decision tree in assessment and management of suicidal patients.

Game theory is a mathematical discipline that attempts to quantify outcomes based on the effect of people's decisions. However, unless we have access to vastly more data and a platform to model interactions with patients, game theory cannot provide numeric descriptions of many real world situations. Limitations include incomplete information, the dynamic nature of interactions, and because human behaviour is not always rational and people do not always act in their own best interests. Nevertheless, modelling the potential effect of decisions made by patients and clinicians by considering the goals, motivations and influence on the other person's decisions might help understand the dynamics that lead to certain outcomes (see Fig. 1). Having access to large bodies of relevant clinical data might in time permit the quantification of decision trees in assessing potentially suicidal patients, and train a clinician-patient model to learn from interactions and assess whether proposed solutions accord with expected outcomes. The vast analytic potential of artificial intelligence drawing on all coded clinical data, and even patterns of conversation in real time, might in time allow a dynamic assessment of the effect of clinical decisions on both risk and resource allocation.

The first task of the clinician is to try to maximise the information on the basis of which an approach to the patient is planned, for example, what is known about the patient's circumstances and motives. The second element is to explore the patient's decision-making processes, in order to consider the effect of any responses by the clinician on incentives that are available and the patient's potential for cooperation with any treatment plan. Utilising game theory in clinical interactions requires the patient to have sufficient awareness of and ability to understand the clinician's perspective to be able to decide on the options that are presented. A third application of game theory is in the analysis of the clinician's interaction with the patient, and whether the behaviour of the clinician or another aspect of the service is impeding a cooperative outcome. A fourth element relevant to suicidal patients is reaching an agreement on treatment that includes a plan for the future and actions that might be taken for both the patient and the service to ensure the plan is followed.

An important part of any strategic interaction is to document the decision-making process and the responses of the patient to any plan that has been made. Clinical notes often include very detailed histories but only brief and vague treatment plans without documented input from the patient. The routine application of elements of game theory in these critical interactions could improve outcomes for patients and clinicians. Rather than looking for increasingly sophisticated risk assessment algorithms on which to base clinical decisions,^14^ clinical software employing artificial intelligence based on the mathematics of game theory that is able to model elements of the interactions among an assessing clinician, the mental health service and a potentially suicidal patient may be of greater assistance to clinicians in developing strategies for the care of suicidal patients.

## About the author

**Olav Nielssen**, MBBS, MCrim, PhD, FRANZCP, is a professor of psychiatry at Macquarie University, Sydney, New South Wales, Australia.
